# Successful Vaginal Delivery after Induction of Labour in a Patient Treated for Non-Hodgkin's Lymphoma of the Cervix: A Case Report and Literature Review

**DOI:** 10.1155/2022/3541046

**Published:** 2022-02-10

**Authors:** Paola Quaresima, Gabriele Saccone, Valeria Zuccalà, Giuseppe Guarascio, Livio Leo, Giuseppina Amendola, Fulvio Zullo, Michele Morelli, Roberta Venturella

**Affiliations:** ^1^Unit of Obstetrics and Gynaecology, “Magna Graecia” University, Catanzaro, Italy; ^2^Department of Neuroscience, Reproductive Science and Dentistry, School of Medicine, University of Naples Federico II, Naples, Italy; ^3^Department of Anatomic Pathology, “Pugliese Ciaccio” Hospital, Catanzaro, Italy; ^4^Department of Radiology, “Pugliese Ciaccio” Hospital, Catanzaro, Italy; ^5^Department of Obstetrics and Gynecology Beauregard Hospital, AUSL Valle D'Aosta, Italy; ^6^Complex Operative Structure Obstetrics and Gynaecology, Annunziata Hospital, Cosenza, Italy

## Abstract

**Objective:**

Primary non-Hodgkin's lymphomas of the cervix are rare; they represent about 1% of all cases. There are no available guidelines regarding the safest mode of delivery after treatment and resolution of a cervical lymphoma. *Case Report*. We report the first case of a successful vaginal delivery after induction of labour in a woman recovered from a primary large B-cell lymphoma of the cervix and a literature review.

**Conclusion:**

In carefully selected patients with fully treated non-Hodgkin's lymphoma of the cervix with no residual disease, induction of labour via prostaglandins pessary may be a safe option if indicated.

## 1. Introduction

Primary non-Hodgkin's lymphomas of the cervix are a rare finding, representing about 1% of all cases of non-Hodgkin's lymphoma [1]. Among different subtypes, large B-cell lymphoma (LBCL) is the most common primarily involving the lower uterine segment [2, 3]. There are no available guidelines on the mode of delivery for women treated for a cervical lymphoma, and few cases have been described about [4–8]. We report the first case of a successful vaginal delivery after induction of labour in a woman recovered from a primary large B-cell lymphoma of the cervix.

## 2. Case Report

**Figure 1:**
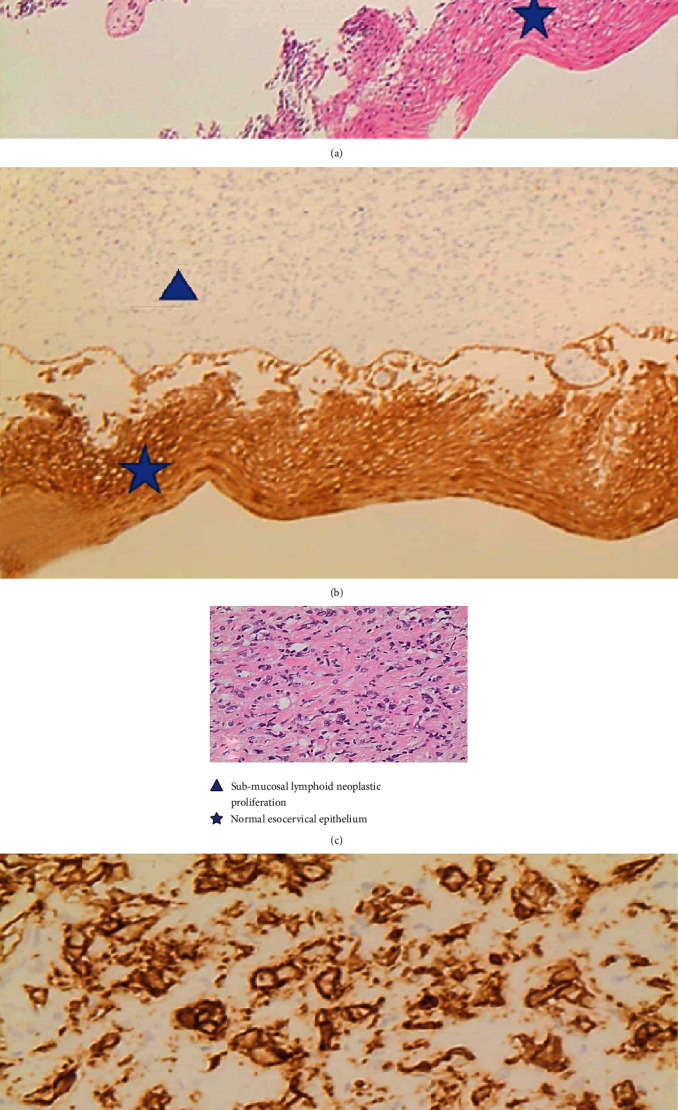
Pathology examination. (a) EE 4x; (b) 4x, immunohistochemical for CK, AE1-AE3: esocervical epithelium is strongly positive; (c) EE 10x, diffuse proliferation of large lymphoid cells with fibrous stromal reaction; (d) 10x, immunohistochemical for CD20: large lymphoid cells are strongly positive.

A 30-year-old nulliparous Caucasian woman presented with a history of dysfunctional uterine bleeding lasting for six months. The patient did not report any past or present significant medical history. Blood analysis was within the normal range, with white blood cells 6000 U/L, haemoglobin concentration 13 g/dL, haematocrit of 33%, and platelet count 165 × 10^3^/*μ*l. The last smear test, performed 24 months before, was negative for neoplasia. At the transvaginal evaluation, the cervix appeared to be irregular due to the presence of a 5 cm diameter mass. The patient underwent hysteroscopy, with a fractional curettage and cervical biopsies. Endometrial and endocervical curettage revealed benign glandular tissue, but cervical biopsies demonstrated a diffuse large B-cell lymphoma (LBCL). The immunohistochemical evaluation of the cervical lesion biopsy was positive for B-cell markers (CD20+, CD10+, BCL6+, and PAX5+), as shown in Figure 1.

**Figure 2:**
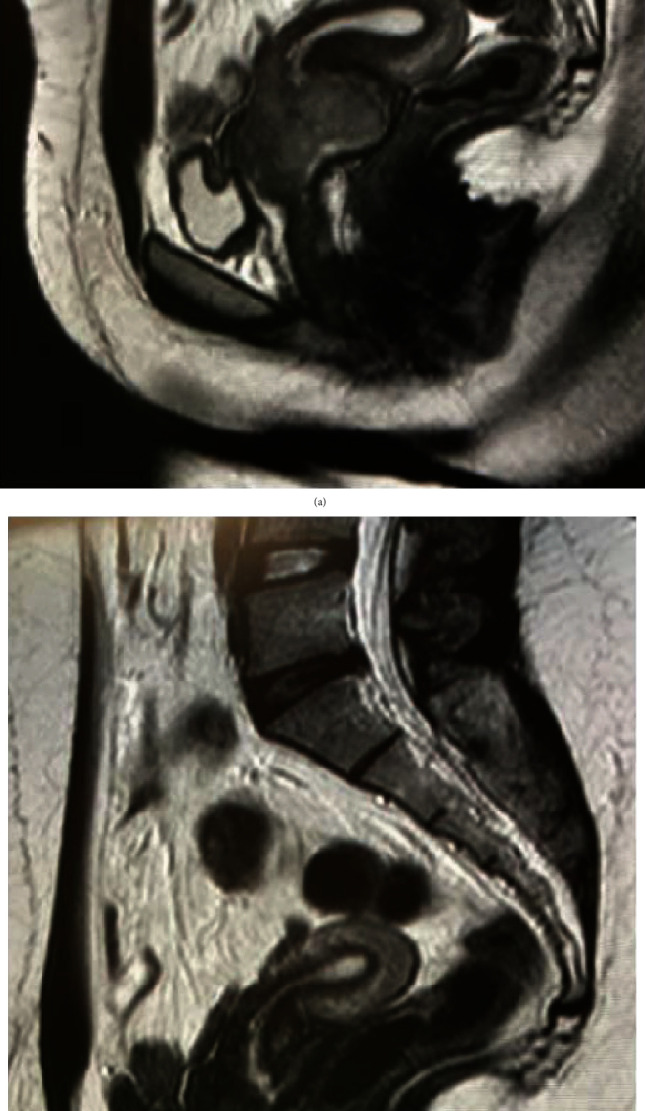
Magnetic resonance T2 weighted images. (a) Low uterine segment appearance at diagnosis. (b) Low uterine segment appearance after treatment.

Both a computed tomography (CT) and a magnetic resonance image (MRI) demonstrated the presence of a cervical mass without extra pelvic lesions. A positron emission tomography (PET) revealed mild uptake of the radioactive tracer at the low uterus level. Following completion of staging studies, the woman was referred to the oncology team, who staged the patient's tumour as IE-B, one extra lymphatic site without fever, night sweats, or weight loss (Ann Arbor staging system). Eight cycle courses of chemotherapy and immunotherapy were recommended. The treatment protocol was based on the combination of: CHOP+Rituximab (Cyclophosphamide, Doxorubicin, Vincristine, Dexamethasone, and Rituximab). Before chemotherapy, depot Leuprolide 11.25 mg intramuscular injection was given every 12 weeks to preserve future fertility against potential chemotherapy's side effects. Six months after completion of medical treatment, a transvaginal ultrasound revealed resolution of the cervical lesion, and follow-up cervical biopsies did not reveal residual disease. PET, CT, and MRI revealed a complete resolution of the cervical lesion. Pre and posttreatment MRI are available in Figure 2. One year after completion of chemotherapy, the patient conceived spontaneously. The pregnancy was uneventful, and at 41 weeks of gestation, according to national guidelines, an induction of labour for postterm pregnancy was offered to the women. Her Bishop score, for cervical evaluation prior to the induction of labour, was calculated and resulted to be 2 (firm, closed, posterior, 50% effaced cervix with a minus two head station); therefore, a Prostaglandin E2 pessary (Propess, Ferring SPA) was introduced into the vaginal posterior fornix. Active labour started at 24 hours from the protocol beginning, and a healthy female of 3100 g was delivered at 36 hours. Cervix dilated and effaced regularly; no cervical trauma was reported. A case report summary is available in Table 1.

## 3. Discussion

Primary non-Hodgkin's lymphomas of the cervical stroma are localized at the level of the cervix without any involvement of the myometrium or evidence for leukaemia at the time of diagnosis [9].

A primary cervical involvement by a lymphoma is a rare finding. Among subtypes, large B-cell is the most common non-Hodgkin's lymphoma of the cervix, representing approximately 31% of cases [10]. Affected women usually present with symptoms such as abnormal vaginal bleeding, dyspareunia, and/or pelvic pain, whereas for fever, night sweats, and weight loss, traditional lymphoma symptoms are uncommon [4–7]. The appropriate management starts with a cervical biopsy with pathological immune-phonotypical evaluation, to differentiate cervical non-Hodgkin's lymphoma from benign and malignant disease of the cervix. Papanicolaou testing is usually of any help in the diagnosis of cervical lymphomas due to the subepithelial location of the abnormal cells [11]. CT and PET are crucial to determinate the extension of disease [12]. Both the FIGO and the Ann Arbor staging systems are used for extra nodal lymphomas [13].

Management with chemotherapy and immunotherapy reduces the need for radiotherapy or surgical resection, particularly for women during childbirth age [14, 15]. Current chemotherapy regimens are safe and can spare fertility, particularly when GnRH agonists (Leuprolide) are used in conjunction with treatment [15–18], although oocyte cryopreservation can be proposed in some cases.

There are no available guidelines regarding the safest mode of delivery after treatment for a cervical B-cell lymphoma. There is occurrence of potential complications such as pelvic outlet obstruction or potential lower uterine segment rupture (due to incomplete resolution of the disease or architectural changes after radiations, let the mode of delivery become an argument of discussion) [4–8]. Indeed, the available literature regarding the mode of delivery for patient affected by a cervical lymphoma reports that only two out of the five previously reported cases experienced a successfully vaginal delivery [7, 8]. One out of three cases delivered via caesarean section received a specific indication due to the previously treated cervical lymphoma [6]. All but one pregnancy occurred at least two years after resolution of the disease, and only one has been diagnosed during pregnancy [5]. Almost all cases have been treated with chemotherapy (4/5), one out of five also received a surgical treatment (cold knife conization) [6], and one also received radiotherapy [7]. No delivery complications have been reported. A description of the five previously reported cases is available in Table 2. Induction of labour has been never described after a treatment for a cervical lymphoma; our case is therefore unique.

## 4. Conclusions

In carefully selected patients with fully treated non-Hodgkin's lymphoma of the lower uterine segment and cervix with no apparent disease, induction of labour via prostaglandins pessary may be a safe option in case of indication.

## Figures and Tables

**Table 1:** Case report summary.

Case report summary
Thirty years old; first pregnancy
Dysfunctional uterine bleeding
Blood analysis: white blood cells 6000 U/L, haemoglobin concentration 13 g/dL, haematocrit 33%, platelet count 165 × 10^3^/*μ*l
Transvaginal evaluation: presence of a 5 cm diameter cervical mass
Hysteroscopy, with a fractional curettage and cervical biopsies: diffuse large B-cell lymphoma of the cervix (CD20+, CD10+, BCL6+, and PAX5+)
Computed tomography-magnetic resonance imaging-positron emission tomography: cervical mass without extra pelvic lesions
Tumour stage: IE-B: eight cycle chemotherapy CHOPS+Rituximab (Cyclophosphamide, Doxorubicin, Vincristine, Dexamethasone, and Rituximab)
Six months after treatment completion: no residual disease
One year after treatment completion: spontaneous conception
Induction of labour with Propess at 41 weeks of gestation: successful vaginal delivery

**Table 2:** Mode of delivery in women affected by cervical lymphoma, literature review.

	Literature review	Mode of delivery	Lymphoma treatment	Cervical lymphoma status
1	Sandvei et al. 1990 [4]	Caesarean section: narrow pelvis	Chemotherapy	Two years after resolution of the cervical lymphoma
2	Wang et al. 1999 [5]	Caesarean section: arrest of cervical dilatation	Unavailable	Lymphoma diagnosed during pregnancy
3	Lorusso et al. 2007 [6]	Caesarean section: cervical lymphoma treatment	Surgery: cold knife conization and chemotherapy	Three years after resolution of the cervical lymphoma
4	Ferreri et al. 2008 [7]	Vaginal delivery	Chemotherapy and radiotherapy	Three years after resolution of the cervical lymphoma
5	Parva et al. 2010 [8]	Vaginal delivery	Chemotherapy	Five years after resolution of the cervical lymphoma
6	Current case 2021	Induction of labour with vaginal delivery	Chemotherapy	One year after resolution of the cervical lymphoma

## Data Availability

The used to support the findings of this study are available from the corresponding author upon request.
